# Cell-Penetrating Peptide Enhances Tafazzin Gene Therapy in Mouse Model of Barth Syndrome

**DOI:** 10.3390/ijms252413560

**Published:** 2024-12-18

**Authors:** Rahul Raghav, Junya Awata, Gregory L. Martin, Douglas Strathdee, Robert M. Blanton, Michael T. Chin

**Affiliations:** 1 Molecular Cardiology Research Institute, Tufts Medical Center, Boston, MA 02111, USA; rahul.raghav@tuftsmedicine.org (R.R.); j_awata@charter.net (J.A.); gregory.martin@tuftsmedicine.org (G.L.M.); robert.blanton@tuftsmedicine.org (R.M.B.); 2 Cancer Research UK Beatson Institute, Glasgow G61 1BD, UK; d.strathdee@beatson.gla.ac.uk

**Keywords:** tafazzin, barth syndrome, gene therapy, cell-penetrating peptide, cardiolipin

## Abstract

Barth Syndrome (BTHS) is an early onset, lethal X-linked disorder caused by a mutation in tafazzin (TAFAZZIN), a mitochondrial acyltransferase that remodels monolysocardiolipin (MLCL) to mature cardiolipin (CL) and is essential for normal mitochondrial, cardiac, and skeletal muscle function. Current gene therapies in preclinical development require high levels of transduction. We tested whether TAFAZZIN gene therapy could be enhanced with the addition of a cell-penetrating peptide, penetratin (Antp). We found that TAFAZZIN-Antp was more effective than TAFAZZIN at preventing the development of pathological cardiac hypertrophy and heart failure. These findings indicate that a cell-penetrating peptide enhances gene therapy for BTHS.

## 1. Introduction

Barth Syndrome (BTHS) is a lethal, incurable, early onset X-linked recessive disorder that affects 1 in 300,000 live births, characterized by dilated cardiomyopathy, muscular hypotonia, and cyclic neutropenia. BTHS is caused by a mutation in the gene encoding tafazzin (TAFAZZIN), an inner mitochondrial membrane acyltransferase that remodels immature monolysocardiolpin (MLCL) to mature cardiolipin (CL), resulting in reduced mature CL and elevated MLCL levels. This alteration in CL composition affects the assembly and normal function of the enzyme complexes present within the mitochondrial membrane that are involved in oxidative phosphorylation, resulting in impaired electron transport chain function accompanied by structural and functional defects in the mitochondria. Striated muscle cells in the heart and skeletal muscle are thought to be susceptible to tafazzin deficiency due to their high energy requirements and abundance of mitochondria to support excitation–contraction coupling. The selective effect on neutropenia is not well understood (reviewed in [1]).

To date, there are no specific disease-targeted therapies available for BTHS patients, and survival beyond young adulthood is rare. Heart failure, often accompanied by shortness of breath, exercise intolerance, muscle weakness, chronic fatigue, and pain can lead to potentially lethal arrhythmia, defibrillator implantation, and cardiac transplantation, which can alleviate heart failure but does not improve skeletal muscle weakness. The current state of therapeutic approaches has recently been summarized [2]. AAV-mediated TAFAZZIN gene replacement therapy has shown efficacy in preserving mitochondrial and cardioskeletal function in a TAFFAZIN knockdown mouse model [3], in restoring mitochondrial function in fibroblasts [4], and in preserving and reversing the heart failure in a knockout model of BTHS when a sufficiently high fraction of cardiomyocyte transduction is achieved (approx. 70%) [5].

We hypothesized that the addition of a cell-penetrating peptide (CPP) may improve TAFAZZIN gene therapy, theoretically, by facilitating the entry of protein shed by the infected cells into the surrounding uninfected cells to propagate and extend the therapeutic benefit. Cell-penetrating peptides are a diverse group of peptides that facilitate the uptake of a wide variety of molecular cargos into cells through a variety of proposed mechanisms, although the predominant mechanism is likely through endocytosis (reviewed in [6]). A previous report indicated that the addition of a CPP derived from HIV TAT protein enhanced gene therapy in a mouse model of Fabry Disease [7]. Here, we report that the addition of a CPP, penetratin, derived from *Drosophila Antennapedia* protein (Antp) [8], enhances TAFAZZIN gene therapy in a knockout mouse model of BTHS, and that the beneficial effect on cardiac function is not solely dependent on improvements in cardiolipin remodeling.

## 2. Results and Discussion

**Figure 1:**
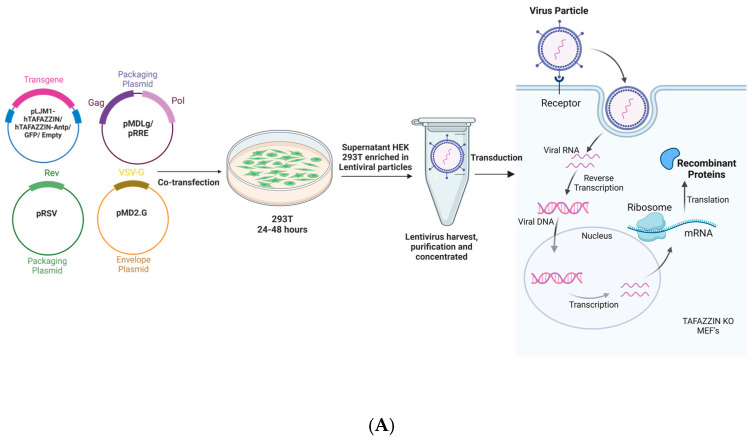

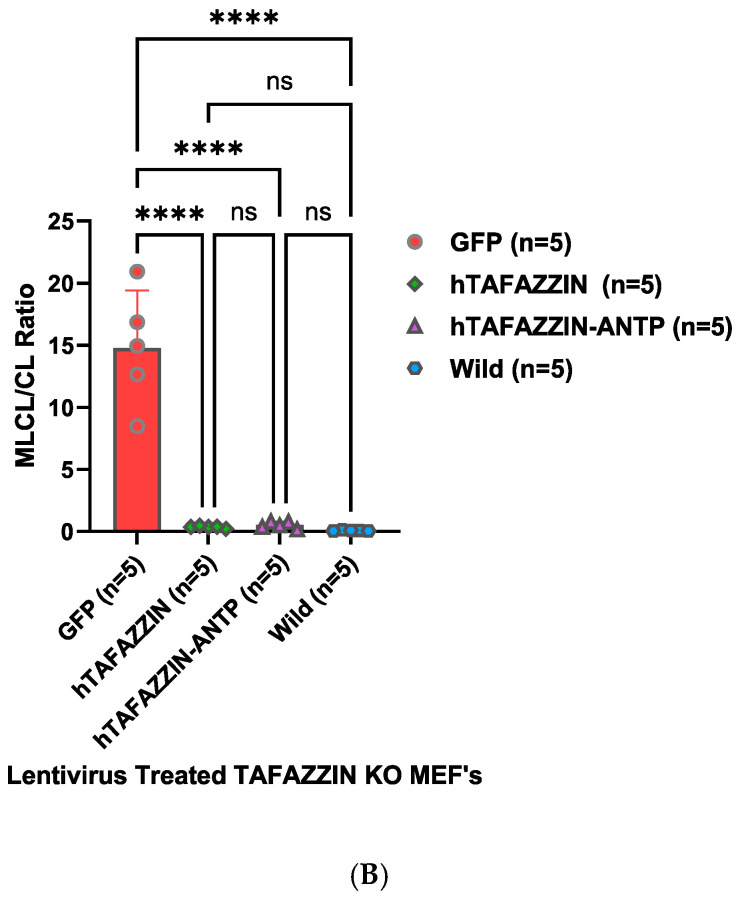
Schematic presentation of 3rd generation lentivirus production and transduction of TAZ KO mouse embryo fibroblasts (MEFs). (**A**) Lentiviral construct containing the gene of interest along with the lentiviral packaging plasmids are co-transfected into 293T cells. Following incubation of cells, supernatant containing lentivirus is harvested, purified, and concentrated. (**B**) MLCL/CL ratio in MEFs after lentivirus treatment. Statistical analysis is performed using IBM SPSS-29 software. One-way ANOVA is conducted between the groups, followed by Bonferonni correction for multiple comparison testing. The results are considered significant when the corrected probability level is <0.05. **** Indicates the significant difference between the Wild/hTAFAZZIN/hTAFAZZIN-Antp and GFP-treated TAFAZZIN KO MEFs at *p* < 0.0001. Nonsignificant differences are indicated by “ns”.

**Figure 2:**
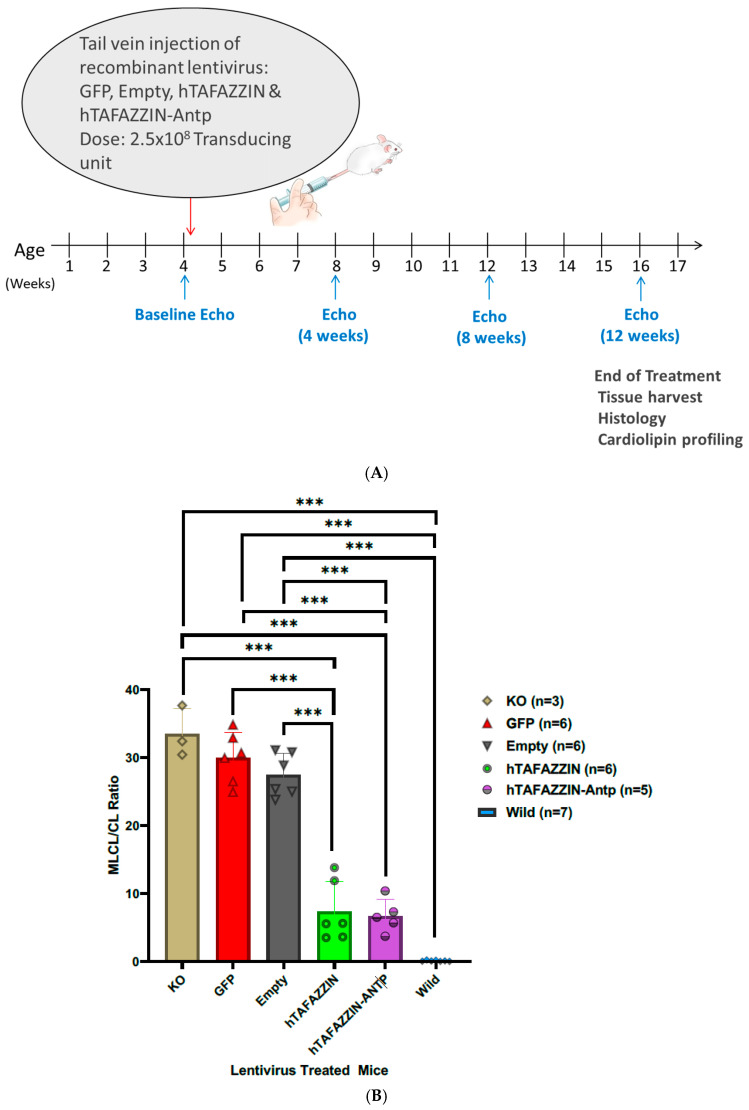

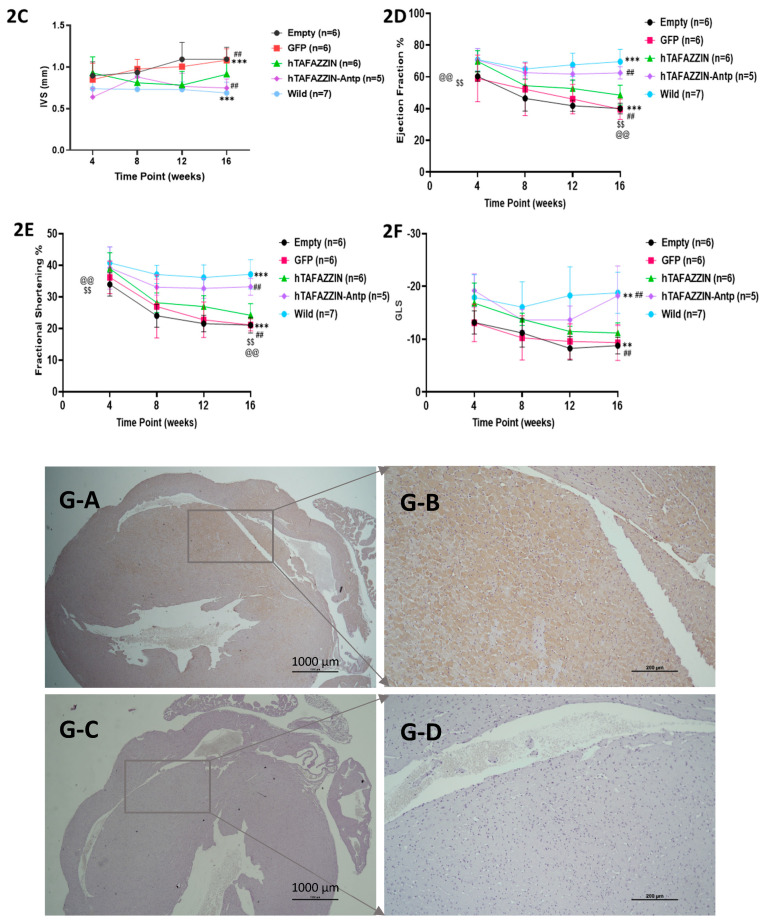
Tafazzin lentiviral gene therapy affects MLCL/CL ratio, cardiac hypertrophy, and cardiac function. (**A**) Schematic illustration of methodology adopted for TAFAZZIN KO mice lentivirus treatment: Baseline echocardiograms (ECHOs) were performed on TAFAZZIN KO mice prior to systemic treatment with a single dose of lentivirus (~2.5 × 10^8^ transducing units) via tail vein injection at 4 weeks of age (hTAFAZZIN, hTAFAZZIN-Antp, GFP, and empty virus). Serial ECHOs were performed every 4 weeks until 16 weeks. After that, mice were euthanized for tissue harvest and downstream analysis. (**B**) MLCL/CL ratio in mouse hearts. There is no significant difference between the KO, empty, and GFP-treated mice but a significant difference was found when we compared the KO/GFP/empty-treated mice with the Wild/hTAFAZZIN-Antp/hTAFAZZIN-treated mice (**C**) Interventricular septum thickness. (**D**) Left ventricular ejection fraction. (**E**) Left ventricular fractional shortening. (**F**) Left ventricular global longitudinal strain. Statistical analysis was performed using IBM SPSS-29 software. One-way ANOVA was conducted between the groups, followed by Bonferonni correction for multiple comparison testing. Within the groups, multiple comparisons were carried out by repeated measures ANOVA with correction by the Bonferroni method. The results are considered significant when the corrected probability level is <0.05 (**/##/@@/$$ *p* < 0.01, *** *p* < 0.001). *** Indicates the significant difference between the wild type and the empty/GFP-treated mice. ## indicates the significant difference between hTAFAZZIN-Antp and empty/GFP-treated mice. In (**C**,**D**), @@/$$ indicates the significant difference between the time points within each group (empty or GFP). (**G**) Photomicrograph A,B show the GFP immuno-positive cells in the heart section of GFP lentivirus-treated TAFAZZIN KO mouse at 20× and 100× total magnification. Photomicrograph C,D show the negative control (no primary antibody) for GFP at 20× and 100× total magnification.

Tafazzin is required for cardiolipin remodeling, and an alteration in the MLCL/CL ratio to greater than 0.3 is considered diagnostic for BTHS, while a reversal of the MLCL ratio is widely considered as a surrogate endpoint for the efficacy of therapy. Initially, to check the effectiveness of lentiviral TAFAZZIN gene therapy in vitro, TAFAZZIN KO MEFs were treated with lentiviruses expressing native hTAFAZZIN, hTAFAZZIN-Antp, and control GFP lentivirus for a week, followed by the LC-MS analysis of the cardiolipin composition (schematically shown in Figure 1A). The LC-MS analysis showed a dramatic and significant reduction in the MLCL/CL ratio to near WT levels for hTAFAZZIN and hTAFAZZIN-Antp but not GFP, with no difference between hTAFAZZIN-Antp and native TAFAZZIN (Figure 1B). These findings indicate that the addition of Antp does not significantly affect the CL remodeling activity of TAFAZZIN. To determine lentiviral TAFAZZIN gene therapy efficacy in vivo, TAFAZZIN KO mice were treated with the same lentiviruses, as well as a virus with no cDNA insert, and heart tissues were harvested at the end of treatment (schematically shown in Figure 2A). Heart tissue from TAFAZZIN KO mice treated with lentivirus encoding GFP or without an expressed cDNA displayed a typical BTHS cardiolipin profile, with low levels of CL and higher levels of MLCL resulting in an elevated MLCL/CL ratio, while the tissue from mice treated with viruses encoding hTAFAZZIN or hTAFAZZIN-Antp showed a significant reduction in the MLCL/CL ratio, thereby ameliorating the defect in cardiolipin remodeling (Figure 2B). Again, no significant difference was seen in the remodeling of cardiolipin by TAFAZZIN when Antp was added, indicating that the addition of Antp has no effect on enzyme activity.

Serial echocardiograms demonstrated the progressive deterioration of heart function in the TAFAZZIN KO mice compared with the WT, when treated with lentiviruses devoid of an expression cassette or expressing GFP, as expected. The deterioration was associated with a significant increase in septal wall thickness (Figure 2C), significant reduction in ejection fraction (Figure 2D), fractional shortening (Figure 2E), and global longitudinal strain (Figure 2F). The immunohistochemical staining of heart sections for GFP revealed detectable expression in the GFP-treated mice but not in the controls (Figure 2G). These findings confirm the viral infection and protein expression implied by the change in MLCL/CL ratio. Surprisingly, the mice treated with native hTAFAZZIN did not show a significant improvement in cardiac function compared to the TAZ KO mice treated with GFP or empty virus, despite an improvement in the MLCL/CL ratio. Treatment with lentivirus encoding hTAFAZZIN-Antp showed not only a comparable improvement in the MLCL/CL ratio but also improved septal wall thickness, ejection fraction, fractional shortening, and global longitudinal strain to values comparable to those observed in the wild type mice. These findings show that an improvement in the MLCL/CL ratio may be necessary but not sufficient for an improvement in the cardiac function. They also show that an improvement in the MLCL/CL ratio to levels below 0.3, the clinical threshold for the diagnosis of Barth Syndrome, is not necessary for an improvement in the cardiac function.

A recent study showed that AAV-TAZ rescued cardiac dysfunction in TAZ KO mice and could reverse LV dysfunction, but durable efficacy was achieved only when a high percentage of cardiomyocytes are transduced [5]. In our study, we show that lentivirally transduced native tafazzin (hTAFAZZIN) and hTAFAZZIN-Antp rescued cardiolipin remodeling equally effectively in vitro and in vivo, but only hTAFAZZIN-Antp was able to rescue cardiac function when administered as a single dose in adolescent mice. These findings indicate that this specific cellular penetrating peptide increases the effectiveness of TAFAZZIN gene therapy, but the mechanism is not yet clear. Systemic lentiviral gene therapy is expected to be transduced at a lower efficiency than systemic AAV gene therapy, and this may explain why the MLCL/CL ratio in the treated TAFAZZIN KO animals, although significantly reduced, is still significantly elevated relative to wild type values. We initially hypothesized that the addition of a CPP may foster spread between the cells; however, equivalent reductions in the MLCL/CL ratio regardless of whether a CPP is present is not consistent with a greater spread between the cells, as this would be expected to reduce the MLCL/CL ratio further than that which was observed without a CPP. It is possible that the spread to adjacent cells is still occurring, but the number of cells affected is not sufficient to alter the bulk MLCL/CL ratio in the heart. Regardless of whether intercellular spread is occurring, our work indicates that a reduction in the MLCL/CL ratio may be necessary but not sufficient for the rescue of cardiac dysfunction. Interestingly, the importance of the MLCL/CL ratio in maintaining mitochondrial function in yeast has been questioned [9]. A potential role for tafazzin in promoting mitochondrial function independent of cardiolipin remodeling, while augmented by Antp, is certainly possible but not consistent with the current pathophysiological model for Barth Syndrome. Our findings are certainly consistent with an alternative model of the role of tafazzin in myocardial function and are thus worthy of further exploration. Our recent study of single nuclei gene expression in the TAFAZZIN KO heart implied the disruption of signaling interactions between adipocytes and cardiomyocytes, for example [10], and that it is possible that the augmentation of gene therapy with penetratin may work through restoring these interactions or some other undiscovered mechanism.

To the best of our knowledge, our report is only the second that has used a cell-penetrating peptide to augment gene therapy [7] and is the first report of use in gene therapy targeting a mitochondrial disorder. Our report raises the interesting possibility of the potential utility of CPPs across a wide variety of gene therapies. It strongly supports the potential for CPPs in augmenting gene therapies as a future treatment for BTHS.

## 3. Materials and Methods

### 3.1. Generation of Lentiviruses

We generated third-generation lentiviral constructs based on pLJM1-EGFP (Addgene, Watertown, MA, USA, cat. #19319) without an insert (Empty Virus), expressing green fluorescent protein (GFP), expressing recombinant human tafazzin (hTAFAZZIN), or expressing recombinant human tafazzin modified to contain penetratin by standard cloning methods. The V2 isoform of TAFAZZIN, lacking exon 5 but retaining enzymatic activity, was used for these studies. All clones were confirmed by sequencing. Lentiviruses were packaged in 293T cells by cotransfection of the pLJM-1 construct with the helper plasmids pRSV-REV (Addgene, Watertown, MA, USA, cat. #12253) and pMDLg/pRRE (Addgene, Watertown, MA, USA, cat. #12251, encoding GAG and POL) as lentiviral packaging vectors, as well as pMD2.G (Addgene, Watertown, MA, USA, cat. #12259) as the VSV-G envelope-expressing plasmid. Packaged lentivirus was concentrated using a Lenti-X Concentrator kit (Takara Bio USA, San Jose, CA, USA, cat. #631231) and titered using a p24 ELISA kit as previously described [11,12].

### 3.2. Infection of TAFAZZIN KO MEF Cells and Assessment of MLCL/CL Ratio

Mouse embryonic fibroblasts were isolated from E13.5 TAFAZZIN KO mice as previously described [13]. One million TAFAZZIN KO MEF cells were infected with 10^8^ transducing units of each lentivirus (native hTAFAZZIN, hTAFAZZIN-Antp, and control GFP) and cultured for one week. Lipids were extracted from the infected MEF cell pellets and assessed for the mature and immature forms of cardiolipin by mass spectrometry as described [14,15]. Briefly, the infected MEF cells were trypsinized, collected by centrifugation and washed twice with PBS (Phosphate buffer saline, pH 7.4). MEF cells were resuspended in 80 µL of PBS, and 40 µL of the cell suspension was spotted onto a Whatman 903 protein saver card. Spots were also spotted with 5 µL of 100 µg/mL solution of Cardiolipin (CL (14:0)_4_ = CL (56:0); Avanti Polar Lipids, Alabaster, AL, USA), which served as an internal standard and air dried. Dried spots were excised, transferred to 2 mL tubes, and then extracted with 1 mL chloroform/methanol solution (1:1, *v/v*). Each extracted sample was vortexed and incubated for 15 min at room temperature in a sonicator bath. The filter paper was removed, and the extract was evaporated to dryness with nitrogen at 60 °C. The residue was reconstituted in 100 µL of acetonitrile/isopropanol/water solution (4.5:4.5:1, *v/v*), was transferred to a sample vial, and then capped. A total volume of 10 μL of lipid extract was injected, and chromatographic separation was achieved on the Agilent 1290 Infinity II UHPLC equipped with ZORBAX Eclipse Plus C18 2.1 × 50 mm, 1.8 µm particle column (Agilent, Santa Clara, CA, USA) at 55 °C. Samples were eluted on a gradient between solvent A (acetonitrile/water [60:40], 10 mM ammonium formate, 0.1% formic acid) and solvent B (isopropanol/acetonitrile [90:10], 10 mM ammonium formate, 0.1% formic acid) at 0.5 mL/min as follows: 0–1 min, solvent B 32%; 1–2 min, solvent B was increased to 70%; 2–8 min, solvent B increased to 80%; 8–9 min, increased to 97% and held at 97% until 13 min. Mass spectrometry was carried out on a 6495 Triple Quad LC-MS (Agilent) in the negative ion electrospray ionization (ESI) mode using MS1scanning, monitoring the different ions or species of MLCL and CL to calculate the MLCL/CL ratio.

### 3.3. Assessment of TAFAZZIN Gene Therapy in TAFAZZIN KO Mice

All animal procedures were performed in accordance with the guidelines of the Institutional Animal Care and Use Committee of Tufts Medical Center. A total of 33 male mice were used in the study, and the TAZ KO mice were treated systemically with a single dose of lentivirus (~2.5 × 10^8^ transducing units) via tail vein injection at 4 weeks of age. The viruses used encoded hTAFAZZIN, hTAFAZZIN-Antp, GFP, and empty virus. Serial echocardiograms (ECHOs) were performed every 4 weeks until 16 weeks, and then the mice were euthanized for tissue harvest and downstream analysis. The hearts were harvested for immunohistochemistry and an assessment of the MLCL/CL ratio as described in Section 3.4 and Section 3.5.

### 3.4. Immunohistochemistry of Lentivirally Infected Mouse Hearts

Hearts were carefully dissected out and fixed in 4% paraformaldehyde in 0.1 M phosphate buffer (PB), pH 7.4. The fixative was changed within 24 h, followed by changes with fresh fixative every day for 5 days, and then every third day for another week. Paraffin blocks were made, and coronal sections of 5 µm thickness were cut and stained for the expression of GFP by standard immunohistochemical techniques.

Briefly, sections were treated with 10 mM sodium citrate buffer, pH 6.0, for antigen retrieval to increase the tissue antigenicity. After three washes with TBS-T (Tris Buffered Saline, pH 7.5, 0.05% Tween-20), the sections were incubated in 10% normal goat serum (PK-6101, VECTASTAIN, ABCKit, Vector laboratories, Newark, CA, USA) for 2 h at room temperature to reduce the non-specific staining. Subsequently, sections were incubated in anti-GFP antibody (1:500, Ab290, Abcam, Waltham, MA, USA) for 24 h at 4 °C temperature, followed by repeated washing. Thereafter, the sections were treated for 4 h with biotinylated secondary antibody (PK-6101, VECTASTAIN, ABC Kit, Vector Laboratories). The primary and secondary antibodies were diluted in the blocking buffer (goat serum in TBS-T) and after washing with TBS-T, the sections were incubated with the avidin–biotin–peroxidase complex (PK-6101, VECTASTAIN, ABC Kit, Vector Laboratories, Newark, CA, USA) for 2 h at room temperature. The binding sites of the antigen–antibody interaction were visualized by using the chromogen diaminobenzidine tetrahydrochloride (DAB). The sections were then air-dried, dehydrated in ethanol, mounted with DPX (Distyrene Plasticizer Xylene), and observed under a microscope.

### 3.5. Assesment of MLCL/CL Ratio in Excised Mouse Heart Mitochondria by Mass Spectrometry

The extracted heart was minced in ~10 volumes of MSHE + BSA (mitochondrial isolation buffer + bovine serum albumin: 5 mM/L HEPES + 210 mM/L Mannitol + 70 mM/L Sucrose, 1 mM/L EGTA, pH 7.4) at 4 °C, and all subsequent steps of the preparation were performed on ice. The minced heart was then disrupted using a drill-driven Teflon Dounce Homogenizer with 3–4 strokes. The homogenate was then centrifuged at 600 g for 10 min at 4 °C to pellet the nuclei and tissue debris. The supernatant was transferred into a separate tube and centrifuged at 8000× *g* for 10 min at 4 °C. After removal of the supernatant, the pellet was resuspended and the centrifugation repeated. The final pellet containing mitochondria was resuspended in a minimal volume of MSHE + BSA, and 40 µL was spotted on a Whatman 903 protein saver card. The Whatman filter paper was then air-dried in a biosafety cabinet and stored at −20 °C. Lipids were extracted from the spotted mitochondria, and LC-MS was used to determine the MLCL/CL ratio, with an appropriate internal CL standard, as described in Section 3.2.

### 3.6. Statistical Methods

All data were analyzed by one-way ANOVA and repeated measures ANOVA with Bonferroni correction for multiple comparison testing, using the IBM SPSS 29 software. Corrected *p*-values were considered significant at a level of *p* ≤ 0.05.

## Data Availability

Data is contained within the article.
